# Nurturing Resilience and Healing from Within: The Impact of an 8-Week Yoga Program on Nursing Students

**DOI:** 10.3390/healthcare13070767

**Published:** 2025-03-30

**Authors:** Beverley Martin, Blake Peck, Andy Davies, Daniel Terry

**Affiliations:** 1 School of Nursing and Midwifery, University of Southern Queensland, Ipswich, QLD 4305, Australia; andy.davies@unisq.edu.au (A.D.); daniel.terry@unisq.edu.au (D.T.); 2 Institute of Health and Wellbeing, Federation University Australia, Ballarat, VIC 3350, Australia; b.peck@federation.edu.au; 3 Centre for Health Research, University of Southern Queensland, Springfield, QLD 4300, Australia

**Keywords:** nursing education, self-care, yoga, stress management, resilience, mental health, nursing students, phenomenology, reflective practice

## Abstract

**Background/Objectives:** Nursing students encounter significant stress due to the demanding nature of their academic and clinical training, negatively impacting their mental health and overall wellbeing. Self-care strategies, such as yoga, have been suggested to effectively manage stress and promote resilience. Despite the growing recognition of the importance of self-care in nursing education, there is limited research on the specific benefits of yoga. This study aimed to explore the experiences and perceived benefits associated with undergraduate nursing students’ participation in an 8-week yoga study. **Methods:** A qualitative study using a hermeneutic phenomenological approach was conducted. Participants were Baccalaureate nursing students from an Australian university. Data were collected through semi-structured interviews and analysed using reflexive thematic analysis. Reporting methods followed the consolidated criteria for reporting qualitative research guidelines. **Results:** Among the 14 students who participated, three main themes emerged: “Me Time”, highlighting the importance of prioritising self-care; “Slowing Down,” emphasising the psychological benefits of yoga; and “Self-Acceptance,” reflecting personal growth and improved self-awareness. Participants reported reduced stress, improved mood, and enhanced physical and mental wellbeing. **Conclusion:** Students who participated in yoga were positively impacted through greater stress management and wellbeing. As nursing students transition into the workplace, the ability to manage stress and maintain mental wellbeing becomes even more critical. The high-pressure environment of healthcare settings can exacerbate stress, leading to burnout and decreased job satisfaction. By incorporating self-care practices such as yoga into their routine, nursing students can develop resilience and coping mechanisms that will benefit them as students and throughout their careers.

## 1. Introduction

Self-care among nurses must form an integral aspect of nurse education design, which includes deep reflective learning skills, while acknowledging the potential complexity associated with clinical experiences [1,2]. It is vital that organisations and educational institutions provide training for nurses in emotional self-management skills, strategies to strengthen wellbeing, and approaches to ensure the long-term mental health of its health professionals is protected [3,4].

Despite the importance of self-care, Slemon et al. [5] concluded that there is little consensus regarding the overarching concept and central meaning of self-care. However, in the context of nursing students, self-care has been conceptualised as an approach to manage stress and as a means of building resilience [6,7]. More broadly, self-care is understood and articulated to be an aspect of holistic nursing practice to ensure a healthy lifestyle, along with achievable activities that are undertaken as a response to managing stress [5]. Within this context, it is this definition that has been used to undertake the study.

Building on this understanding of self-care, the goal of Baccalaureate nursing education is to prepare students as generalist registered nurses to practise within an evolving healthcare environment, which is complex, fast-paced, and stressful [8,9,10]. A study by Andrews et al. [11] argued nurses live with uncertainty due to constant changes and pressure within their work environments. A business-orientated approach to healthcare delivery was seeking to manage a ‘broken’ system, which was having a destabilising effect among nurses and impacting their capacity to self-care while compromising self-compassion [11]. Further, it has been argued training to become a nurse is equally stressful due to the demanding nature of their academic and clinical training, negatively impacting student resilience, mental health, and overall wellbeing [12,13]. While other health professions and fields of study, such as medicine and physiotherapy, also experience significant stress, nurses are uniquely challenged due to the emotional demands of patient care. In addition, nursing students must also meet the academic demands of such a role. In order to manage stress and burnout while undertaking training, nursing students must be carefully supported and nurtured as they develop their clinical knowledge and skills in preparation for the workplace [1].

To address these challenges, it remains imperative for those who educate and train nursing students to provide them with the capacity and skills to develop self-management associated with emotional wellbeing. Additionally, addressing inter-personal stressors such as staff conflict, bullying, incivility, and physical aggression should be included in Baccalaureate training [3,14]. In exploring strategies for integrating stress management into the classroom, one approach that has been useful throughout students’ professional training is reflective practice [15]. Reflective practice, which embodies self-reflection, encompasses the engagement of neurobiological and cardiovascular systems from an embodied physiological perspective [16]. Self-reflection is a process whereby students expand mental concepts of first impressions and feelings, to think more deeply about experiences, thereby connecting their knowledge and broader experience [17,18]. The benefits of self-reflection include improved communication, developing meaningful relationships with others, increased self-awareness, and helping students in their personal and professional development as a nurse [19,20].

Furthermore, Pai [21] suggests greater self-awareness and insight were predictors of greater clinical competence among nurses, while being more likely to improve the quality of work undertaken and overall patient outcomes. In addition, self-reflection practices have been demonstrated to assist nursing students to become aware of their stress levels during clinical practice. As such, it has been argued Baccalaureate education programs should include reflection competencies and self-care strategies within the curricula [22]. Sellman [22] indicated that a tension exists between the need for self-reflection, which requires time, space, and deep thinking, and the practical necessity of maintaining the continuity of the curriculum.

Despite these recognised benefits, there remains a significant gap in how undergraduate nurse education programs prepare students for the emotional, psychological, and physical challenges they will encounter as they enter the workforce. One such approach that has shown promise is contemplative pedagogy, which is gaining recognition in higher education and may be useful, specifically in training health professionals [23,24,25,26,27]. Contemplative practice is defined as a set of metacognitive exercises that quieten the mind in order to cultivate a personal capacity for deep concentration and insight [28]. Contemplative pedagogy can act as a counterbalance to a range of pedagogical positions in Western higher education, with an emphasis on connection and awareness.

In support of this approach, in a study by Cunningham and Cayir [29], resilience retreats were developed at the University of Virginia Medical Centre (UVA) for healthcare professionals and also including nursing students undertaking training through the UVA school of nursing. The purpose of the retreats was to provide day-long training to develop the capacity of health professionals to engage in mindfulness practices, such as yoga, and self-efficacy associated with mindfulness. This aligns with the goals of contemplative pedagogy by providing healthcare professionals with the tools to manage stress and enhance their self-awareness. Therefore, it is vital for students to continually take steps towards self-care and personal consciousness, which can be facilitated through contemplative practices and resilience training. While resilience is often acquired through life experiences, practices such as mindfulness, which includes yoga, can support the development of resilience by enhancing self-awareness and emotional regulation.

The relationship between yoga and mindfulness has been well-documented in the existing literature. For example, Schuver and Lewis [30] found that a mindfulness-based yoga intervention effectively managed depressive symptoms and ruminative thoughts in women with depression, indicating that yoga incorporates mindfulness principles to achieve psychological benefits. Similarly, Silva et al. [31] demonstrated that yoga practices inherently involve mindfulness components, such as focused attention and awareness. These findings support the classification of yoga as a mindfulness practice. Furthermore, the holistic approach of yoga, which includes physical postures, breathing exercises, and meditation, fosters a resilient mindset by promoting overall well-being and stress management [32]. While some argue yoga may not directly lead to resilience within a short timeframe, it is important to consider the cumulative effects of yoga. Over time, these benefits can contribute to building resilience by enhancing self-awareness and emotional regulation [32]. To advance self-care principles in nursing education, and foundational to best practice outcomes and healthy work environments, incorporating a variety of activities and practices at different levels is essential [5,13,23,33]. Despite this, current nursing curricula often fall short in preparing students for the multifaceted challenges they will face, and a notable lack of structured self-care practices, such as yoga and mindfulness in healthcare settings, is evident in the literature [34,35]. Contemplative pedagogy, which includes mindfulness and yoga, has shown potential in higher education but remains underutilised in nurse training. Addressing this gap requires research into how yoga can support nursing students’ mental health and overall wellbeing. Therefore, the aim of this study is to explore the experiences and perceived benefits associated with undergraduate nursing students’ participation in an 8-week yoga program.

The objectives of this study are

- To explore the experiences of nursing students participating in an 8-week yoga program.
- To identify the perceived benefits of yoga on nursing students’ mental health and overall wellbeing.
- To assess how yoga practice influences nursing students’ resilience and self-care practices.

The hypothesis of this study is as follows: Participation in an 8-week yoga program will positively affect nursing students’ mental health, wellbeing, and resilience.

## 2. Materials and Methods

A qualitative study informed by a hermeneutic phenomenological approach was conducted to address the aims of the study and investigate the experiences of Baccalaureate nursing students’ participation in an 8-week yoga program. The focus was to understand deeply the phenomena of yoga as it was experienced by the participants within their bodies, as phenomenology is theoretically consistent with the idea that each participant will experience the world and yoga in unique ways, and that each participant is then able to share that experience with the researcher [36]. The central aim in hermeneutics is how to understand the conceptual texts of the participants from the perspective of the historicity of the researcher. The possibility then arises that when we understand each other, we come to a fusion of horizons [37]. Reporting methods followed the consolidated criteria for reporting qualitative research (COREQ) guidelines [38].

### 2.1. The Yoga Program

The Hatha yoga program lasted for eight weeks and was offered as pre-recorded asynchronous online classes which would enable students who were studying the opportunity to participate in the program at a time and place most convenient to their schedule. The class format lasted for 40–45 min of asana flows, which are specific movements and shapes made with the body, and included 5–10 min of shavasana, which are restorative postures, at the end of the class. The yoga instructor was an Iyengar trained senior yoga teacher in the Hatha yoga tradition. The Iyengar tradition is predominately a form of modern postural yoga and offers a systematic set of instructions for teaching various postures [39,40]. Iyengar yoga is unique in its use of props such as blankets, blocks, and straps which allow students of all levels to work safely in the classical yoga asanas (postures). It was anticipated that all participants were at the beginner level and therefore the depth of understanding regarding yoga needed to match this understanding and practice capacity. During the eight-week intervention, the yoga sequencing and approach was intended to give the participant an experience of a comprehensive asana practice that promotes overall wellbeing, stress reduction, and relaxation. Students had only one opportunity to participate in the program.

### 2.2. Participants

**Figure 1:**
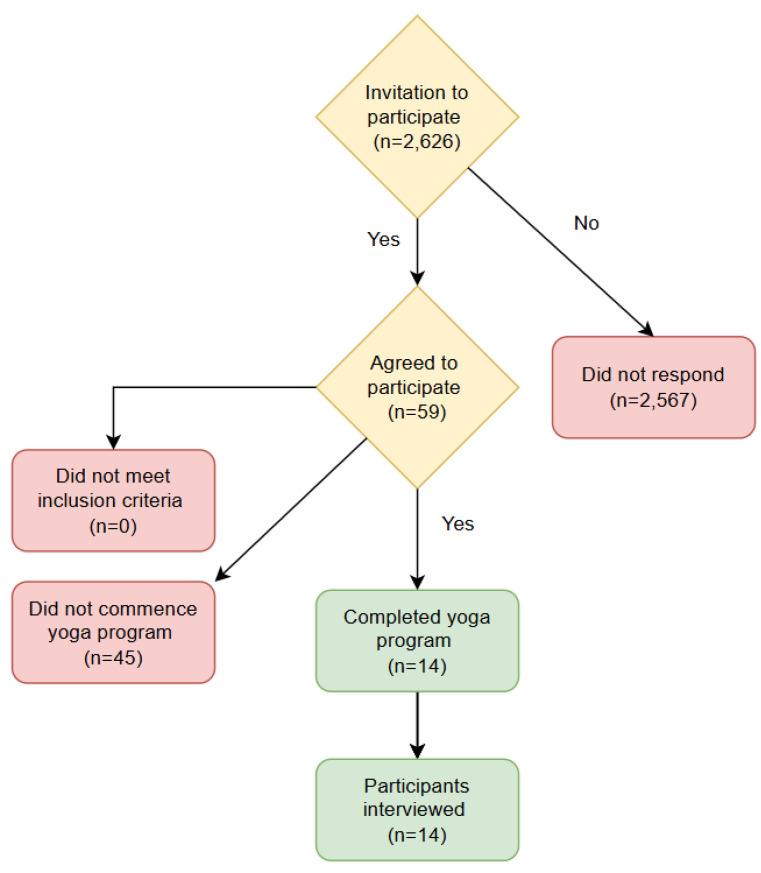
Flow diagram of participation.

All nursing students within the Baccalaureate nursing program at an Australian university, with campuses in metropolitan, regional, and rural areas, were invited to participate in the yoga program (n = 2626). Across the sample of respondents who expressed interest, all were invited to participate in the study (n = 59). To be eligible to participate in the study, participants needed to be medically fit to participate. Students were excluded if they were pregnant, had an injury that would make it difficult to safely undertake key poses as part of the yoga program, or who had already completed the program previously (Figure 1).

### 2.3. Data Collection

The program was run twice with the initial program being run in the second semester of the 2023 academic year and again in the first semester of the 2024 academic year. An invitation to participate in a follow-up one-on-one interview occurred four weeks after the conclusion of each 8-week yoga program. One researcher (BM), specialising in yoga and wellbeing research conducted the interviews using telephone and videoconferencing technology. Each interview lasted between 30 and 60 min, with field notes taken during and after the sessions. Each interview began with an open question inquiring into the experiences of the nurse student following participation in the 8-week yoga program. The semi-structured interview questions were guided by a set of preliminary questions in addition to how the participant responded to previous questions. For example, an initial question asked of participants was “What have you been able to integrate from the yoga intervention into your academic and personal life?” The response may have then led to unstructured questions such as “Were you able to work with your restrictions, such as stiffness in the body?” (Table 1). Interview questions were shared and discussed with healthcare and healthcare workforce experts for clarity, revision, and testing prior to undertaking participant interviews.

### 2.4. Data Analysis

All audio- and video-recorded interviews were transcribed verbatim into Microsoft Word and the data were labelled based on code and the year the interview was conducted. Once the data were transcribed and cleaned, participants were invited to verify the accuracy of their transcripts through member checking. Although the interview transcripts were shared with participants, no participants provided additional comments, corrections, or insights.

The data analysis relied on the reflexive thematic analysis approach as outlined by Braun and Clark [41]. As such, the researcher’s role in knowledge production is at the centre of this approach and pre-existing themes are not specifically set out by the dataset but are achieved through analysis, which also requires a deep reflection approach by the researcher as they engage with the data [41]. Initially, transcripts were read several times and involved looking for key elements across the datasets which in this case were the participant interviews. The purpose was to find repeated patterns of meaning. The data were coded into small ‘chunks’ of meaning as these could represent the initial codes found within the data [41]. The coding process requires the researcher to continuously reflect on and question their assumptions while interpreting the data. This was followed by a deep and prolonged data immersion, thoughtfulness and reflection, active and generative [41]. Braun and Clarke’s method was chosen for its flexibility and emphasis on researcher reflexivity, allowing for a rich, detailed analysis that aligns well with the study’s goals of exploring complex, subjective experiences [41].

Meanings were then assigned to each dataset, and significant quotes from the interviews were initially grouped broadly before being categorised into themes independently by four researchers (BM, DT, BP, and AD). These themes were further refined through collective discussion and consensus within the research team. The themes were named to capture their essence, using participants’ excerpts to enhance confirmability. This systematic approach to data analysis was chosen as it does not consider previous research outcomes and minimises researcher bias, thereby improving the trustworthiness of the research [40]. Data were labelled by providing a code to each participant and the year the participant was interviewed (for example, BT, GK, and HK).

### 2.5. Ethical Considerations

Ethical approval for the study was provided by the Federation University Human Research Ethics Committee (Reference #2023/108) and University of Southern Queensland Human Research Ethics Committee (Reference ETH2024-1102). Details of the study including the purpose, data protection, and storage were provided to all participants prior to the commencement of the 8-week yoga program. Informed consent was obtained from all participants prior to respondents participating in the study.

## 3. Findings

All participants who agreed to participate in the yoga program and follow-up interviews (n = 14) were female, 57% (n = 8) were single, 64% (n = 9) were from a regional centre, 50% (n = 7) were in their second year of study, and while no specific age group was overrepresented, it is noted that those who were aged 30–39 were less likely to participate (Table 2). In addition to participant demographics, within the dataset, three themes emerged and came to represent the experiences of the students who engaged with the online yoga over the eight-week period. The themes included Me Time, Slowing Down, and Self-Acceptance which are discussed in detail.

### 3.1. Me Time

The theme ‘Me Time’ came to represent the importance of making time for yoga practice, putting yourself first, and to make your own health a priority. The idea that ‘getting out of your head’ and ‘into your body’ had the effect of creating calmness within the body and made a difference to the way some participants think. The participants who had family responsibilities expressed being so busy as to feel overwhelmed. This was expressed by a mature-aged student with family responsibilities as follows:

*Feeling overwhelmed felt like having a big knot inside of me. I get snappy towards people and have a short fuse. After each class this became less and I began to have a routine, Thursday gym, sauna and yoga, that was good me time. If I do two hours of uni work on the computer I can justify ‘me’ time with the mat. (BT)*

Finding time for the yoga practice required becoming more self-disciplined in order to do the practice each week and not just go to bed at the end of a long day working with clients. This required pushing through the feelings of exhaustion at the end of each day and was expressed as

*I put a reminder on my phone to allow myself to do the yoga practice before going to bed which usually around 9.30 pm rather than just flop down in the evening. (GK)*

The participants in this study reflected on how they were able to integrate what they had learned from yoga. They expressed the need to take more responsibility for themselves and to find something that worked for them. Changes had been made to fixed routines as the commitment to the eight-week yoga classes required finding something extra within themselves, in an already hectic schedule, particularly during clinical placements. This was expressed by a 20-year-old female student as

*Burning out, here we go again and drained from it. Rehab [clinical placement] was exhausting as it required hoisting patients into equipment and included some patients who can be abusive. Physical fitness [through yoga] helped to destress and relax. I found my mood improved from being down and returned to my happy and cheery self. (EM)*

It was necessary to find strategies that support the wellbeing of nursing students. The physical aspect of yoga was one of these strategies as it developed physical strength and self-discipline. One participant, a 29-year-old nursing student, explained having a good exercise routine helped regulate the emotional side of her life. This realisation had occurred during COVID-19 and where state government mandated the restricted movement of people. This restricted movement of no more than 5 km from a place of residence or ‘forced lockdown’ experience was an opportunity to do something for herself. The forced lockdown had been a time of reflection and greater perspective whereby decisions were made at the time to never ‘let go’ of having an established exercise routine. This was expressed as follows:

*I have never been a naturally fit person and battled with my weight my whole life. I realised that if I am going to continue to do this job [nursing], I need to be physically healthy. I need to take more time out during the day, thirty minutes each day to relax the mind. (GK)*

In addition, due to the nature of modern healthcare services, the importance of protecting the psychological wellbeing of health professionals was identified to respond to yoga. Mindfulness practices were often embedded in physical practices such as yoga and an approach for increasing awareness and responding skilfully to mental processes. As a way of changing fixed mindsets, it was necessary to give the brain a rest from the intellectual and physical demands of nursing which included caring for people. This was expressed as follows:

*When I first started yoga, I noticed how much difference it made to the way I think. There was a massive change in me after the 8-weeks of yoga which included losing weight. I was happy that it was on-line, and this was my space, my time and not worried about body shaming. I could actually relax and take time for myself, after the class it was like OK ready for another day. (TR)*

For nursing students who were working in supportive aged care, their roles were physical as elderly clients need to be showered and have them set for the day. Some of the issues included keeping boundaries between the client and the carer (nursing student) and maintaining awareness as to how the client could still remain independent. This was expressed as

*It can be a challenge when you come across a person who is heavier set, or with Dementia or Parkinsons. These conditions are physical and a challenge to get them up and going as well. Issues of maintaining independence when showering and take responsibility for themselves as much as possible. (GK)*

### 3.2. Slowing Down

In addition to Me Time, the second theme ‘Slowing Down’ emerged and related to the more psychological aspects of yoga. This was how the student nurse participants came to be more in tune with their inner feelings, recognising a need within themselves to become more aware of their own inner energy and levels of exhaustion that occur from a nursing culture that is heavily focused on caring for people. It was important to focus on themselves as well. This was particularly acute for participants who were more sensitive and described themselves as introverted personality types. For example, one participant spoke about this feeling as an outward expression when they said

*I have become a lot more emotionally aware as well. I ask myself what am I feeling here, what emotions are coming up and how do I deal with it in the healthiest way possible. There is a need to make an emotional shift for each client that I see on a daily basis and that takes a lot out of me, and I get quite tired. I have to meet both the physical and emotional needs of my clients as many people are living by themselves and are quite socially isolated. (GK)*

The participants described juggling emotionally challenging environments that included work, family responsibilities that included caring for a sick parent, a child who is unwell, and the responsibilities of being a single parent. This emotional balancing was described by a mature-age student when they said

*I learned to slow down a little bit. I have many personal responsibilities, especially caring for a sick parent and a small child who is unwell. I can’t find the answers. I go what can I do for you guys. When you do not have the answers, you feel like where should I go. If the course (yoga) was not there, I would have had a nervous breakdown. (NS)*

The participants described their reasons for participating in the study which included finding ways to work with the body in releasing the stress that was accumulating in the body from the physical and psychological demands of being a nurse. A participant described this stress as being in a particular area of her body when she stated she carries stress in her shoulders, making upper body stretches essential. She found yoga helped her relax and focus on her body’s signals. At 50, she understood the importance of self-care, particularly as she worked in aged care, and sees many residents struggle to get up. However, she also noted that

*I actually hate exercise with a passion, especially going to a gym, but I found yoga was different. (KO)*

Nevertheless, the student stated she enjoys the mental benefits of yoga, such as relaxation and concentration, and appreciates the time to notice their breathing, heartbeat, and pain.

Furthermore, the importance of breaking the cycle of constant busyness required slowing down in order to find space and learn how to break tasks and responsibilities into chunks. Previously, the academic mindset was to just keep going even if nothing much was penetrating the brain. A mature-aged participant reported more perspective and balance with competing priorities during the semester as follows:

*Sitting at a computer and reading you start to go around in circles. When I did the yoga video, I found that I came back with a fresh headspace. It felt like I was reading a new piece of work. I found mistakes in my work and had new ideas as well. I learnt how to prioritise and shuffle. Two hours on the computer, one hour for me. Two hours on the computer, cook dinner. I would not have made these changes prior to the yoga intervention. (BT)*

### 3.3. Self-Acceptance

The third theme ‘Self-Acceptance’ was an experience of coming to greater awareness and understanding of themselves. For nursing students who participated in the yoga, aspects and pre-constructed layers of who they thought they were began to loosen as the yoga had the effect of deconstructing fixed constructs about themselves. The commitment to the eight-week program created experiences to be with their bodies in ways not previously known. The postures required a greater inner perspective to be reflective and how to work with the instructions given by the yoga teacher. Participants learned more peaceful ways of being more present with themselves. A mature-age student expressed her journey of inner discovery as a single person as follows:

*I decided to make my weight loss a priority, which I am feeling positive about. Life has changed a lot in the last two years in that my children have left home. Trying to cook for one person meant I was buying too much and eating too much. The yoga freed up my mind to think more analytically, cooking for one was where I needed to be and learning to eat appropriate size meals. Doing the yoga, I felt that I was doing something for my body that was good for me. (JP)*

In searching for ways to change fixed patterns of behaviour, there had been changes to what was always leaving things to the last minute, especially submitting assignments. A mature-age student with young children expressed this as follows:

*In the last few weeks I have found myself mentally calmer and thinking clearly and to start managing all the jobs I have put off for the past eighteen months. I have the mental space to look at my HECS debt and try to understand it. Whereas before I was so overwhelmed and felt like it was too much. I feel like this weight has lifted and I have the mental ability and head space to start processing. (BT)*

The physicality of yoga meant that bodies were changing and becoming more flexible, stronger, and toned. This had the effect of being able to see a new version of the physical body as the yoga began to give back to each participant something that made them feel good about themselves. This change in body shape and becoming stronger was described by one participant as follows:

*I developed muscles, loss of weight and toning as well. I could look at myself in the mirror and go there is a difference. I started to like myself. You need to like yourself before you can let other people in. I have come very far in a short space of time considering I have had thirty years of negativity around me. I am doing things I never thought I could do, like becoming a nurse. (TR)*

There were physical restrictions for some mature-age participants that included being diagnosed at an early age (10 years) with a scoliosis. This had the effect of restriction in the body, especially on the side where the scoliosis was located. It was found that more than ever, working towards greater flexibility was possible, even though it was not perfect or thought of by younger people as a practice that is all about stretching. This was described by a 51-year-old first-year fulltime nursing student with family responsibilities as

*It is really important for students to have something else to focus on besides study, work, family and the everyday challenges. Mature aged students have a better appreciation of the importance of wellbeing as many already work in healthcare. In first-year baccalaureate nursing, anatomy and physiology is a year-long subject. Opportunities to demonstrate the benefits of yoga could be included in this subject as demonstrated by younger and mature-age yoga teachers together as each representing, the benefits of remaining flexible. (DB)*

Overall, the theme of ‘Self-Acceptance’ highlighted how yoga helped nursing students gain a deeper understanding of themselves. The eight-week program allowed them to deconstruct pre-constructed layers of their identity and be more present with their bodies. Participants reported feeling more peaceful and reflective, with one mature-age student noting significant personal growth and improved self-care.

## 4. Discussion

This qualitative study aimed to explore the experiences and perceived benefits associated with undergraduate nursing students’ participation in an 8-week yoga study. It emerged from the experiences that students’ education and training was found to be stressful and required juggling many competing priorities and responsibilities. These findings are consistent with the wider literature regarding nursing student experiences within education [1]. Participants reported several benefits from the yoga practice, including reduced stress levels, improved mental clarity, and enhanced overall well-being. These results suggest that incorporating structured self-care practices, such as yoga, into nursing curricula could be a valuable strategy for supporting students’ mental health and resilience [14,42,43].

In this study, the theme of ‘Me Time’ highlighted the importance of making time during the week for self-care. The experiences shared by participants showed multiple factors that led to decisions to find more balance in their lives. The primary feelings expressed were visceral, constantly exhausted, and unable to break the cycle of constantly being busy. The cohort who participated in this study were more mature adults and carrying significant personal responsibilities which included the academic study load, family responsibilities, and working, which was essential to financially support the current cycle of transition. This was highlighted in the wider literature, whereby reconciling demands of family responsibilities and household chores added to feeling overloaded and more vulnerable to stress for female students [44]. The importance of maintaining resilience was necessary; however, constant busyness and limited time and energy were barriers to maintaining optimal health. This is similar to the wider literature whereby constant exhaustion was a visceral feeling and contributed to stress and anxiety and it was necessary for students to schedule regular time for body and mind, and recharge through practices such as yoga [1].

The foundation for teaching self-care practices among nurses begins within university education [14,45]. Mindfulness training should form part of curricula education as there has been a significant correlation between resilience and mindfulness among healthcare professionals [13,45]. Specific regular weekly practices could include mindfulness and yoga exercises in which students begin to expand their consciousness, enhance resilience, and work with themselves both physically, emotionally, and psychologically [13,24,25].

The second theme ‘Slowing Down’ was identified as a higher order function of self-care and related to both physical and psychological wellbeing. Within the study, participants described searching for knowledge and understanding as to why slowing down could be an opportunity for cultivating greater self-awareness. Practices that restore our ability to feel ourselves include body-centred forms of meditation, contemplative practices, and yoga therapy practices [46]. Some participants expressed being at a point of not having any answers for the difficulties they were encountering and an opportunity to participate in yoga meant opening to a place within themselves that intuitively they knew would benefit their body and mind. In this sense, to understand why slowness is a skill to be cultivated required a reorientation inward of listening to and feeling the body. Among participants, they were looking for transformation of themselves beyond the daily routine of caring for patients, and yoga provided this opportunity whereby control and surrender are necessary when creating the postures.

Whether the participants knew this at the outset of the intervention is not important; they had made a commitment to transformation, including resistance to change, which has been highlighted in the seminal work of Kramer [47]. According to Kramer [47] individuals participating in yoga will not be able to remain the way they currently experience themselves. It has been stated that they either become more crystalized and rigid or they break out of pattens and transform. This takes place through generating energy within the system and using the postures as a tool to explore and open the body. This way of working has the effect of slowing down the mental processes which students had found overwhelming and instead use the postures as a more nuanced action to open the body. The participants expressed mindsets and beliefs about themselves that were conditioned and difficult to change.

The final theme ‘Self-Acceptance’ meant that bodies were changing and going back to the old version of themselves was no longer an option. The possibility of liking oneself began to emerge from a deep place inside themselves, which contrasts with years of negative thoughts which impacted self-esteem. In a study by Sudheer et al. [48], the influence of yoga on ‘Gunas’ (personality) and self-esteem revealed yoga has greater influence on holistic personality growth when compared to routine physical exercise. Therefore, the knowledge obtained from a deeper understanding of oneself revealed that the nature of personality is not a fixed construct. In addition, through effort and resilience, the yoga had given back to each participant some part of themselves that had been previously lost and searched for. Finding time each week for a yoga practice was an experience in slowing down to make time for the practice, with benefits including personal growth, resilience, and letting go of the fixed perception of the self [49].

To advance nurse education, it is necessary to consider the characteristics of nursing programs. The educational dynamics and focus must be on the learner as a whole person, who actively participates in learning in order to develop capacities for reasoning, reflection, self-management, and self-regulation [50]. In a study by Cooper et al. [2], students assigned value to being included in an inclusive learning environment and which supervisors are underprepared and report students concerns. Therefore, a broader base of knowledge and therapeutic presence including building relationships between faculty and students would mutually afford the meaning of wellbeing and self-care for staff and students. Contemplative pedagogy is an emerging field of research and an appropriate alignment for sustainable health practices in nurse education. A core subject in this field would offer opportunities to develop students’ understanding for optimal health and self-care.

Other reasons for amplified levels of stress in healthcare were related to clinical placements and supervision. A study by Levett-Jones et al. [51] reported novice Australian nursing students experienced a feeling a fear of making mistakes. Further, less positive experiences may form a barrier to learning such as fast-paced environments on the students’ first day of clinical placement [52,53,54] and emotional environments that are highly charged [55]. It would therefore seem critical that nursing students understand the importance of good psychological wellbeing and that educational systems carry this responsibility to educate nursing students on the importance to self-care strategies. The findings from the study indicate that undergraduate curricula may benefit from an emphasis on contemplative pedagogy as an essential component for wellbeing including skills learned in preparation for the workforce. In adopting contemplative practices, it had been demonstrated that participating in yoga promotes self-awareness and self-reflection for nursing students that leads to a decrease in stress and anxiety and cultivates increased self-understanding.

Protecting the current and future wellbeing of students is a responsibility of nurse education institutions as nursing has been identified as a stressful profession [56]. Work-related stress affects both the individual and organisations worldwide and is negatively associated with patient outcomes [57]. One of the main arguments in the literature concerns the impact of burnout and stress on the physical and mental health of the individual, with everyday situations including death and dying being significant stressors for nursing staff [26,54,58]. Mareno-Martinez and Sanchez-Martinez [59] indicate work satisfaction is a crucial factor that can enhance or undermine levels of work engagement for health professionals. Further, Mareno-Martinez and Sanchez-Martinez [59] found that health workers who have more work overload have a propensity to be more committed to their work. These findings highlight the importance of teaching students to find and cultivate balance within their lives, nurtured during Baccalaureate training. These skills include having a physical practice such as yoga, making time for self-care, and reflective practices of slowing down that enable the parasympathetic system to come more into balance [60]. These skills must be considered as competencies essential for self-care and wellbeing, and contribute to the physical, psychological, and emotional wellbeing of student nurses [24,26].

Incorporating yoga and self-reflection practices into nursing education not only benefits students during their studies but also equips them with essential skills for their professional careers [61]. As new nurses enter the workforce, they face complex and high-stress environments that can lead to burnout and high attrition rates. By fostering resilience and self-awareness through practices such as yoga, nursing graduates are better prepared to manage stress, maintain their mental health, and provide high-quality patient care [62]. With up to 30% attrition rates globally among new nurses within the first five years, integrating these practices into nursing curricula could be a crucial strategy for supporting new nurses and addressing this ongoing issue [63]. Ultimately, these practices help to create a more sustainable and effective healthcare workforce, benefiting both the individual practitioners and the healthcare system as a whole [29].

Evidence from previous studies supports the benefits of yoga and self-reflection practices in reducing stress and improving mental health among nursing students and professionals. For example, key researchers [24,25,42,43,64] found that nursing students who participated in a yoga program reported significant reductions in stress and anxiety levels. Practical implementation of these practices can include integrating them into existing courses, offering workshops, or providing access to resources and support. Personal testimonials from nursing students who have benefited from these practices highlight their positive impact on mental health and resilience. Policy recommendations for nursing education institutions and healthcare organisations include supporting the integration of yoga and self-reflection practices into curricula and providing ongoing support for these practices in the workplace.

## 5. Future Research

This study builds on previous research demonstrating the benefits of yoga for managing stress, anxiety, and depression [65]. However, future research should further investigate self-care practices inspired by contemplative neuroscience and yoga in undergraduate nurse education. Future studies should also address specific research questions, such as the long-term effects of yoga and self-care practices on nursing students’ mental health, resilience, and professional performance. Further, it should include diverse populations of nursing students, including those from different cultural backgrounds, to understand how these practices might be adapted to various contexts and to ensure the findings are generalisable. In addition, comparative studies are needed to evaluate the effectiveness of different self-care practices, such as yoga, mindfulness, and other contemplative practices, to determine which are most beneficial for nursing students. Future research should explore the best strategies for implementing self-care practices in nursing curricula, including identifying potential barriers and facilitators to successful integration. It is vital to understand the impact of nursing students’ self-care practices on patient care outcomes, to demonstrate the broader benefits of these practices for the healthcare system. Lastly, longitudinal studies are also a potential avenue to observe the long-term benefits of self-care practices among nursing students as they transition into professional practice, so as to understand how these practices influence their career longevity and job satisfaction.

Currently, there is a gap in the research regarding the importance of self-care for nurses in Baccalaureate education. Many nurse education programs do not have a sustainable health practice unit. Developing such a unit would involve incorporating various forms of learning. These include silence (disconnected from knowledge), received knowledge (listening to the voices of others), subject knowledge (the inner voice), procedural knowledge (separate and connected knowing), and constructed knowledge (integrating the voices) [66]. A goal of the program would be to expand nurses’ self-knowledge, self-awareness, and decision-making skills. This would be achieved by using subjective knowledge based on personal needs and what is required to sustain wellbeing. The results would seek to highlight the impact of introducing a sustainable health practices unit into the nursing curriculum.

## 6. Limitation

The results of this study provide insights into short-term benefits of a single eight-week yoga program. This study was executed with limited resources and the results may not be sustainable unless students participate in regular yoga practice over time. Limitations to the study include the fact that it was conducted at one university setting.

A key limitation was the timing of the research. Although COVID-19 was officially declared ‘over’ in Australia on 20 October 2023, there was a backlog of student clinical placements due to the pandemic, which impacted future placements. Initially, 59 students signed up for the yoga intervention. However, to expedite the clinical placement program, semesters were condensed from twelve weeks to six weeks. This created an academic overload, preventing students from continuing their commitment to the yoga program.

Another limitation was the small number of participants. Despite sending out several rounds of invitations and contacting students who initially expressed interest, there was a lack of interest that precluded the capacity to recruit more participants along with time constraints. Further limitations include self-selection bias, as participants were self-selected and may have had a pre-existing interest in yoga or higher motivation to improve their wellbeing.

Lastly, it is acknowledged that participating in the first or second semester may influence students differently due to variations in academic demands. For instance, the first semester often involves students adjusting to new courses and schedules, which may affect their ability to fully engage with the yoga program. Conversely, the second semester may bring increased academic pressures, such as end-of-year exams and assessment deadlines, potentially impacting students’ participation in the program. These variations should be considered a limitation of the study and limit the understanding of the effectiveness of the program.

## 7. Conclusions

The present study revealed the experiences of nursing students who participated in an 8-week yoga program designed for advancing self-care principles in Baccalaureate nurse training. The study identified that the stress levels for student nurses were amplified due to responsibilities of study, caring for family, and participation in the workforce. Students who participated in the study were searching for ways to break the cycle of constant busyness and feelings of always being exhausted. Due to the nature of nursing, caring for people was found to impact the physical, psychological, and emotional levels of the body. The nurses in this study expressed insights as to how yoga had assisted them to slow down and make time for their own self-care. The effect of yoga was to realign the energetic field of the body which then brought renewed energy, a freshness throughout the system, and a feeling of increased wellbeing. Importantly, this research has found that contemplative practices, which include yoga, assist students to think deeply and reflectively about themselves. The benefits of participation included enhanced life meaning and purpose, finding more space within the body for self-care, and cultivating a mindset necessary for personal growth.

Incorporating these practices into nursing curricula not only benefits students during their studies but also equips them with essential skills for their professional careers. As new nurses enter the workforce, they face complex and high-stress environments that can lead to burnout and high attrition rates. By fostering resilience and self-awareness through practices such as yoga, nursing graduates are better prepared to manage stress, maintain their mental health, and provide high-quality patient care. Further, integrating these practices into nursing curricula could be a crucial strategy for supporting new nurses and addressing this ongoing issue. Ultimately, these practices may help to create a more sustainable and effective healthcare workforce, benefiting both individual practitioners and the healthcare system as a whole.

## Figures and Tables

**Table 1:** Semi-structured interview schedule.

	Interview Questions
1.	You have been attending a yoga class for eight sessions, what has been your experience of being in the class? (Prompt questions guided by the answer) - You mentioned the internal dialogue from your mind as you made the effort to participate in the classes. Can you explain this more? - Your experience has highlighted the need to look after yourself on a regular basis. Can you explain this more?
2.	What have you learned from your experience so far, has it been positive and what aspects of the class have you enjoyed or have been challenging? (Prompt questions guided by the answer) - Did you find similarity with regard to the postures that were taught by the yoga instructor? - What compelled you to want to do the yoga practice on a regular basis?
3.	With regard to the postures, what have you learned from making the shapes and were you able to follow the instructions from the teacher? (Prompt questions guided by the answer) - You mentioned the breath work more than anything else assisted you to get into the postures. Can you explain this more? - You mentioned becoming more emotionally aware as you participate in the yoga movements. Can you explain this more? - You refer to needing to slow down a lot more as your life was becoming overwhelming. Can you explain this more?

**Table 2:** Participant demographic characteristics.

Code, Year	Area Living	Age Range (Years)	Gender	Year of Study	Marital Status	Children	Study Mode
GK	Regional	50–59	Female	Third	Single	No	Fulltime
NS	Regional	18–29	Female	Second	Single	No	Fulltime
GM	City	40–49	Female	Second	Married	Yes	Fulltime
HK	-	50–59	Female	Second	-	-	Part-time
ER	City	30–39	Female	Second	Single	No	Fulltime
DB	Regional	50–59	Female	First	Single	Yes	Fulltime
TR	City	30–39	Female	First	Married	Yes	Fulltime
AP	Regional	18–29	Female	First	Single	No	Fulltime
JP	Regional	30–39	Female	First	Married	Yes	Fulltime
KO	Regional	50–59	Female	Second	Single	-	Part-time
RK	City	30–39	Female	Third	Married	Yes	Fulltime
EM	Regional	18–29	Female	Second	Single	No	Fulltime
BT	Regional	18–29	Female	Second	Single	No	Part-time
KH	Regional	18–29	Female	Third	Married	Yes	Part-time

## Data Availability

Dataset available on request from the authors.
